# Single versus double row suture anchor fixation for greater tuberosity fractures – a biomechanical study

**DOI:** 10.1186/s12891-017-1868-7

**Published:** 2017-12-01

**Authors:** Gernot Seppel, Tim Saier, Frank Martetschläger, Johannes E. Plath, Alberto Guevara-Alvarez, Julia Henschel, Martin Winkler, Peter Augat, Andreas B. Imhoff, Stefan Buchmann

**Affiliations:** 1 Department of Orthopedic Sports Medicine, Klinikum rechts der Isar, Technical University Munich, Ismaninger Str. 22, 81675 Munich, Germany; 2 Department of Orthopedics and Trauma Surgery, Krankenhaus Barmherzige Brüder, Munich, Germany; 3 Department of Reconstructive Joint Surgery and Sportstraumatology, Berufsgenossenschaftliche Unfallklinik Murnau, Murnau, Germany; 4 Center for Shoulder and Elbow Surgery, ATOS Clinic Munich, Munich, Germany; 5 Department of Trauma Surgery, Klinikum Augsburg, Augsburg, Germany; 6 Institute of Biomechanics, Berufsgenossenschaftliche Unfallklinik Murnau, Murnau, Germany; 7 Institute of Biomechanics, Paracelsus Medical University, Salzburg, Austria; 8 Orthopädisches Fachzentrum, Weilheim, Germany

**Keywords:** Greater tuberosity fracture, Suture anchor, Arthroscopic, Double row fixation, Single-row

## Abstract

**Background:**

Fractures of the humeral greater tuberosity (GT) are a frequent injury progressively treated with arthroscopic suture anchor repair. Yet, no biomechanical study has been performed comparing fixation strength of arthroscopic single- (SR) vs. double row (DR) fixation.

**Methods:**

Standardized fractures of the greater tuberosity were created in 12 fresh frozen proximal humeri. After random assignation to the SR or DR group the fixed humeri were tested applying cyclic loading to the supraspinatus and infraspinatus tendon. Load to failure and fragment displacement were assessed by means of an electrodynamic material testing machine using an optical tracking system.

**Results:**

Load to failure values were higher in the DR group (649 N; ±176) than in the SR group (490 N; ±145) however without statistical significance (*p* = .12). In greater tuberosity displacement of 3–5 mm surgical treatment is recommended. The fixing constructs in this study did not reach displacement landmarks of 3 or 5 mm before construct failure as shown in previous studies. Thus the applied traction force (N) at 1 mm displacement was analyzed. In the SR group the load at 1 mm displacement was 277 N; ±46 compared to 260 N; ±62 in the DR group (*p* = .65).

**Conclusion:**

The results suggest that both techniques are viable options for refixation of greater tuberosity fractures.

Level of Evidence: Laboratory study.

## Background

Isolated fractures of the greater tuberosity represent a common injury accounting for up to 1/5th of all proximal humeral fractures [1, 10]. Due to the limited dimension of the subacromial space even small residual superior displacement may cause clinical impairment [2, 9]. It has been shown, that conservative treatment for dislocated greater tuberosity fractures is associated with pain and impaired range of motion (ROM) [8]. Therefore, especially in patients with high functional demands surgical treatment is recommended if superior dislocation of the greater tuberosity reaches 3-5 mm of displacement [4, 9]. Gerber and Warner described that malunion is one of the most common complications following surgical treatment of proximal humeral fractures [9]. As a general rule, it is well known that bony healing depends critically on anatomical reposition and rigid retention [31]. Besides, mechanical stability is essential to allow early functional rehabilitation in order to achieve satisfying clinical results [15]. Arthroscopic treatment of greater tuberosity fractures is an established method [12, 28, 29]. Previous biomechanical studies demonstrated a secure retention and reposition using suture anchors [3]. However, none of theses studies investigated on the influence of the postero-superior rotator cuff (M. infraspinatus (ISP) and M. supraspinatus (SSP)) forces on the stability of greater tuberosity fractures that were treated arthroscopically using suture anchor techniques. Further, to the knowledge of the authors, there is no biomechanical data comparing single vs. double-row anchor fixation.

The aim of this biomechanical study was to compare knotless suture anchor reconstruction in greater tuberosity fractures using a single- vs. double-row fixation. Therefore, a biomechanical human cadaveric in vitro model was established, that incorporated forces of the postero-superior rotator cuff on the greater tuberosity.

The primary hypothesis of this study was, that double-row fixation confers greater load-to-failure strength and less secondary displacement under cyclic loading compared to single-row fixation of the greater tuberosity.

## Material & Methods

This biomechanical study was approved by the institutional review board of the Technical University Munich, Munich, Germany.

### Specimen preparation

**Fig. 1:**
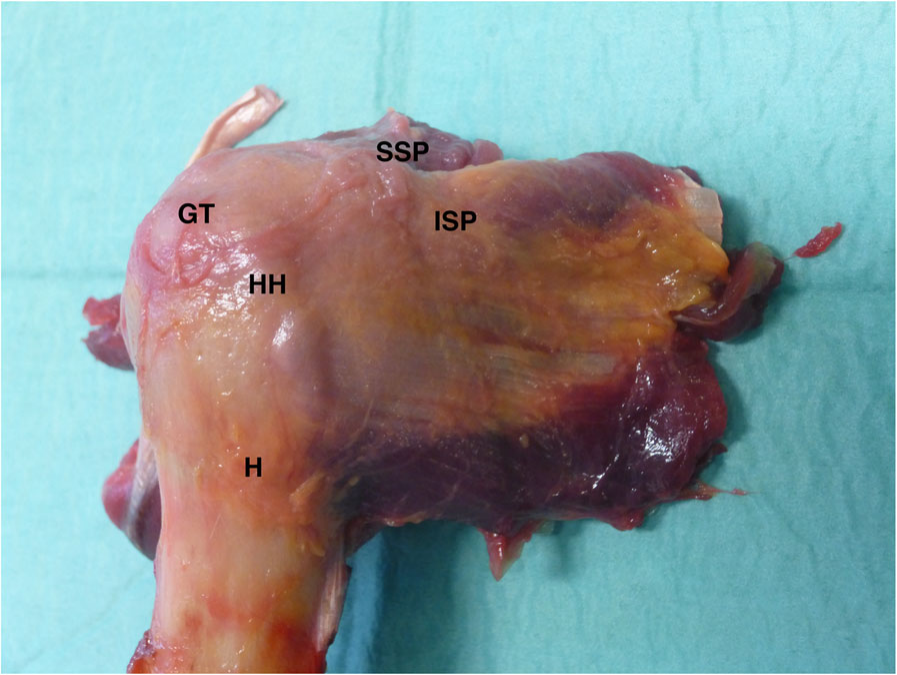
Specimen; (H: Humerus; HH: Humeral Head; GT: Greater Tuberosity; SSP: Supraspinatus; ISP: Infraspinatus)

Testing was performed using 6 paired (right vs. left) fresh-frozen human cadaveric shoulder specimens (6 male cadavers; mean age, 61,3 years; age range, 45 to 68 years). Specimens were thawed at room temperature 24 h before testing. The humeri (with completely preserved Mm. supraspinatus and infraspinatus) were dissected from the specimens after disarticulation and removal of all other soft tissues (Fig. 1).

For inclusion, several criteria were mandatory: (1) intact supero-posterior rotator-cuff; (2) BMD > .50 g/cm^2^; (3) no history and/or signs of previous fracture, and (4) cadaveric age younger than 70 years.

Bone mineral density (BMD) measurement (dual-energy x-ray absorptiometry [in grams per square centimeter]) was performed for each specimen to guarantee consistent BMD values between groups (mean BMD, 0.65 g/cm^2^; range, 0.58 to 0.67 g/cm^2^).

The distal condyles were removed and the humeral shaft was adjusted to a standardized length of 20 cm. A standardized greater tuberosity fracture was created in a 50° degree angle to the humerus shaft using a chisel as described by Lin et al. [17].

### Biomechanical testing

**Fig. 2:**
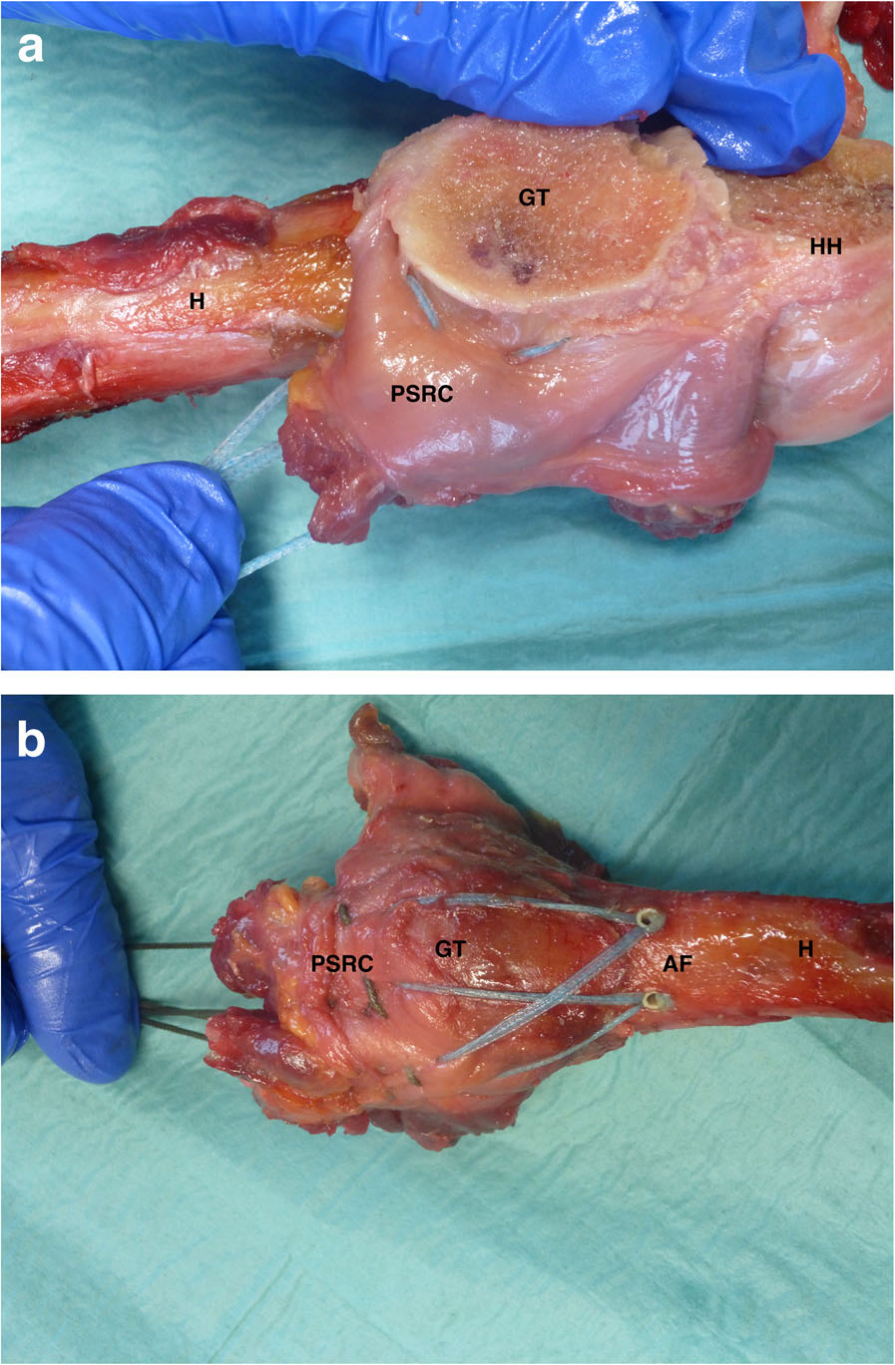
**a**-**c**: Single Row Fixation; (H: Humerus; HH: Humeral Head; GT: Greater Tuberosity; PSRC: Postero-superior Rotator Cuff; AF: Anchor Fixation)

**Fig. 3:**
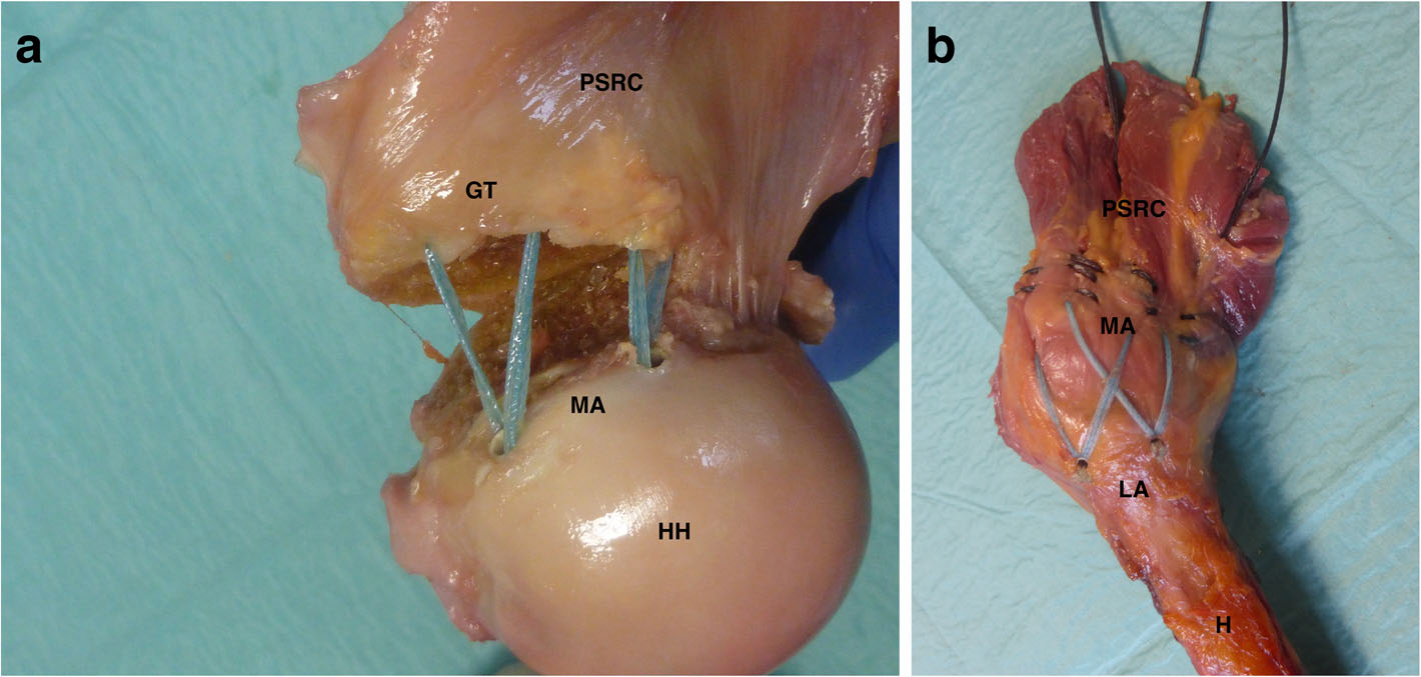
**a**-**b**: Double Row Fixation; (H: Humerus; HH: Humeral Head; GT: Greater Tuberosity; PSRC: Postero-superior Rotator Cuff; MA: Medial Anchors; LA: Lateral Anchors)

After anatomical reposition and temporary fixation using 2 mm K-wires paired specimens (right vs. left) were randomly assigned to the following groups (each *n* = 6) by lottery:

(1) Single-row suture tape (FiberTape, Arthrex Inc., Naples, USA) reconstruction using knotless anchor fixation with two anchors (PEEK SwiveLock 4,75 mm, Arthrex Inc., Naples, USA). Using Mason-Allen-stiches SSP-tendon was armed 1.5 cm from the anatomical footprint and the superior ISP-tendon 1 cm medial from the footprint with two suture tapes. In the tension direction of the SSP and according to the manufacturer instructions the two suture tape loaded anchors were placed laterally and 5 mm distally to the fracture into the humeral head (Fig. 2 a, b).

(2) Double-row suture tape (FiberTape, Arthrex Inc., Naples, USA) reconstruction using knotless anchor fixation with four anchors (PEEK SwiveLock 4,75 mm, Arthrex Inc., Naples, USA). First 2 suture tape loaded anchors were placed medial to the fracture at the cartilage-bone-transition-zone. At the identical anatomical sites as described above the tendons of SSP and ISP where perforated and the tapes where shuttled through the tendons, then the suture tapes were crossed. In the direction of movement for the SSP according to the manufacturer instructions two suture tape loaded anchors were placed laterally and 5 mm distally to the fracture into the humeral head. The anchors insertion sites were prepared by a 4 mm drill. (Fig. 3a and b)

All tests were performed at room temperature, and the surface of the specimens was constantly kept moist using isotonic saline solution.

**Fig. 4:**
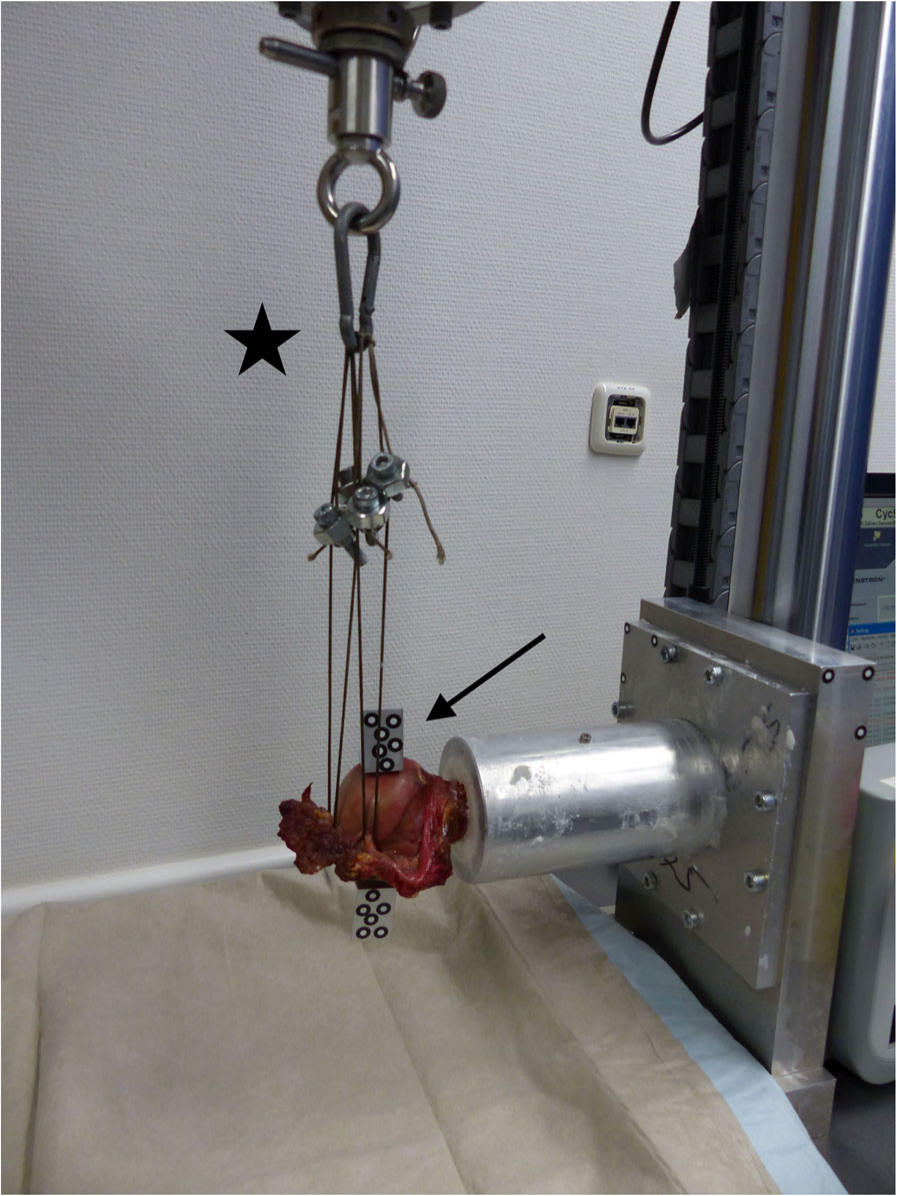
Mechanical Testing Setup

To perform biomechanical testing, the humerus shaft of each specimen - adjusted to a standardized length of 20 cm - was potted and rigidly fixed in casting resin (RENCAST FC53; Huntsman Advanced Materials, Bergkamen, Germany) using a custom-made jig [3, 17]. Optical markers were in line with the line of force and rigidly attached to the humeral head and greater tuberosity to measure relative movements between fragments using an optical tracking system (Pontos, GOM, Braunschweig, Germany). This system recorded displacement with an accuracy of .025 mm. The specimens were then fixed to an electrodynamic material testing machine (E3000 with Instron Dynacell -Measuring range +/−3Kn-; Instron Ltd., High Wycombe, United Kingdom)(Fig. 4).

During mechanical tests, the humeral shaft was fixed at 0° of abduction. To simulate abduction a braided, abrasion-resistant wire (Black Cat Power Leader Rhino, Tostedt, Germany) was used to reinforce the SSP and ISP tendons. The ends of the wire were fixed to the mechanical testing machine to apply an axial tensile load. The force was distributed equally between SSP and ISP tendons (50:50). The muscle tendons were preloaded with 50 N. Starting with 50 N load level for cyclic loading the force was increased by 40 N every 1000 cycles until failure.

Ultimate failure load [N], along with displacement [1 mm], and mode of failure were recorded for all specimens. Failure was defined as fracture of the humeral neck, anchor loosening, suture rupture or complete dislocation of the greater tuberosity.

### Statistical analysis

A post hoc power analysis using G*Power (version 3.1.9.2; Franz Paul, Kiel, Germany) was performed to determine the power of the study. On the basis of the results of the Fisher exact test, an effect size of 0.85 was calculated. With this effect size, an a of .05, and the sample size of 6, a power of 0.80 was calculated. For statistical analysis, SPSS software (version 22.0; IBM, New York, NY) was used. Normal distribution was tested and confirmed with the Kolmogorov- Smirnov test. Quantitative parameters are given as means, standard deviations, and 95% confidence intervals. To evaluate the differences regarding BMD, specimen age, and load to failure between groups, a 1-way analysis of variance with a Tukey post hoc test was used. The Fisher exact test was used to analyze the failure modes between the testing groups. A significance level of *p* < .05 was accepted as a statistically significant difference.

## Results

There were no significant differences between groups concerning BMD, morphology, and age (n.s.).

**Fig. 5:**
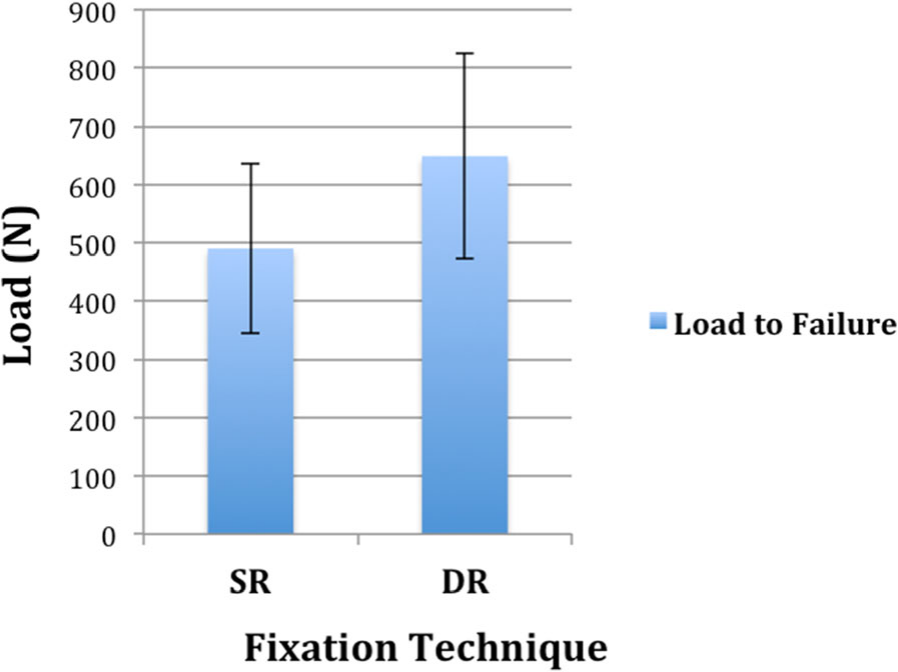
Load to failure: Comparison of ultimate failure loads for single and double row fixation of greater tuberosity fractures (*p* = .12). Data are presented as mean and standard deviation (N)

Regarding the mean cyclic load to failure (LtF) of suture anchor reconstruction in standardized fractures of the greater tuberosity, there were higher values in the DR group (649 N; ± 176) compared to the SR group (490 N; ± 145). (Fig. 5).

However, no significant difference could be seen between groups (*p* = .12).

In the SR group the mean number of cycles to failure were 10,782 ± 4203 whereas construct failure could be observed in the DR group at 14317 ±4450 cycles in average.

During the testing it was recognized that a 5 mm displacement – as defined in prior studies [3, 4, 9, 17, 25] - could only be achieved in one fourth of all specimen before construct failure. Thus, it was preferred to measure the applied force (N) at 1 mm displacement to receive objective and comparable results between the groups. In the SR group the mean applied load for 1 mm displacement was 276.7 N ± 46.2 whereas in the DR group an average load of 259.5 N ± 61.8 could be observed to achieve 1 mm displacement.

**Fig. 6:**
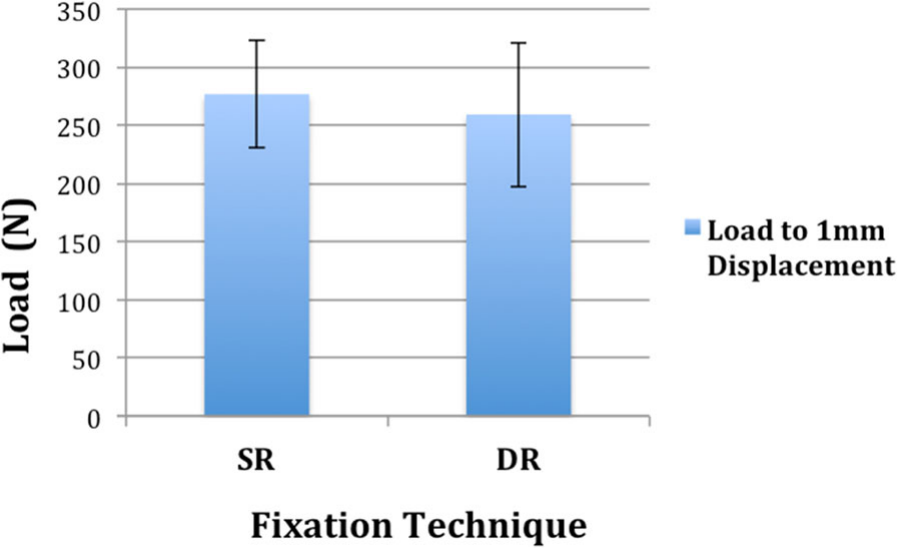
Displacement: Comparison of loads for 1 mm displacement (mm) for single and double row fixation of greater tuberosity fractures (*p* = .65). Data are presented as mean and standard deviation (N)

However, there was no difference regarding the loads for 1 mm displacement between single and double row fixation of greater tuberosity fractures (*p* = .65) (Fig. 6).

### Mode of failure

In the SR group the constructs failed because of fracture at least 2 cm distal to the humeral surgical neck in three cases (50%) whereas a pull-out of the anterior anchor of the greater tuberosity could be seen in one specimen (16,6%). In the DR group three specimens (50%) failed due to fracture at least 2 cm distal to the humeral surgical neck. A pull-out of the anterior medial anchor of the greater tuberosity could also be seen in one specimen (16,6%). A rupture of the abrasion-resistant wire fixation occurred in two specimens (33,3%) at ≥650 N in the SR and the DR group, respectively. Therefore, there was no evident difference regarding failure modes between groups.

## Discussion

In this biomechanical in-vitro study of arthroscopic suture anchor fixation techniques for greater tuberosity fractures, there was no significant difference between single-row and double-row repair regarding load to failure and applied traction force for 1 mm fracture displacement. Thus the studies primary hypothesis must be rejected. To the best of our knowledge, this is the first study presenting biomechanical data on single- vs. double-row knotless suture anchor repair of greater tuberosity fractures.

Treatment modalities of greater tuberosity fractures include conservative treatment, open, minimally invasive percutaneous, and arthroscopic procedures [9, 11, 13, 16, 17, 24, 26, 27]. It has been shown that nonoperative management reveals disappointing results in case of displaced fractures of the greater tuberosity [26]. Thus surgical treatment is advised if superior displacement of ≥3-5 mm occurs to avoid malunion of the fractured greater tuberosity.

During the last years the focus has moved towards arthroscopic techniques [6, 12, 14, 16, 23, 24] due to the benefit of reduced pain, soft tissue damage and skin incision. Additionally arthroscopy allows to inspect the entire glenohumeral joint as well as to address concomitant pathologies in one session [20]. Besides, arthroscopic suture anchor repair using knotless implants may reduce operative time, simplifies the procedures and waives suture knots. One of the benefits of suture fixation is that it depends on the strength of the rotator cuff tendons themselves and not only on the bone quality.

Furthermore, this technique seems to be superior to other rigid methods like screws regarding load to failure and load to 5 mm displacement [17]. This represents an important recognition as fractures of the greater tuberosity are commonly comminuted and usually seen in young and active patients [1, 18]. Furthermore, in case of comminuted fragments of the greater tuberosity or in older patients with osteopenic bone [10] a wider contact surface of refixation could be helpful regarding healing and stability [30]. In addition, no additive surgery to remove osteosynthesis material is needed.

A gradual increase of greater tuberosity displacement that requires intervention – usually described as 3-5 mm displacement [3, 4, 9, 17, 25] – could not be seen in this study.

Neither in the single-row nor in the double-row group all specimens consequently reached these two greater tuberosity displacement landmarks before construct failure.

This could be explained by an increased stability of the fixation method compared to rigid systems like two cancellous screws and due to the fixation of the entire tendinous insertion of the postero-superior rotator cuff (SSP/ISP). To receive objective and comparable results between groups, the applied traction force (N) was analyzed at 1 mm displacement (277 N (SR) vs. 260 N (DR)). Consequently, no fixation technique (SR vs. DR) showed significant advantages over the other. In this study medial row of the DR technique was not tied as knotless suture tape systems were used. This may have affected the 1 mm displacement that occurred more easily in the double row repair than the single row repair.

In their biomechanical study Lin et al. [17] described a 3 and 5 mm displacement of the greater tuberosity in the double-row suture anchor group at 262.5 N and 370.3 N load as well as in the suture-bridge technique at 321 N and 398.5 N load. In the two-screw fixation group a 3 and 5 mm displacement was apparent at a load of 186.7 N and 249.2 N, respectively.

This shows that the applied traction forces for 1 mm displacement are similar to the loads described by Lin et al. [17] for 3 mm displacement.

The ultimate failure loads in the study of Lin et al. [17] were 480 N (Double-row), 493.3 N (Suture-bridge) and 340 N for the two-screw fixation. The present study showed comparable failure loads in the SR group (490 N) and even higher loads in the DR group (649 N).Regarding failure modes, a fracture of the humeral surgical neck was noted in 50% of both groups indicating that the construct itself was stronger than the native bone. Lin et al. [17] also described a fracture of the humeral surgical neck as common failure mode. They also found a dislocation of the anterior anchor in one specimen of the Suture Bridge group.

In the present study, dislocation of a suture anchor was noticed in only one specimen (16,6%) per group.

A rupture of the abrasion-resistant wire fixation occurred twice (33,3%) in each group at more than 650 N in our study. This high load of failure underlines the findings of very stabile fixation methods – single- and double row. However, both fixation constructs are expected to tolerate significantly higher loads than the maximal supraspinatus force of ~302 N [5]. This might be explained by the fixation method. In this study the common footprint of the postero-superior rotator cuff in line with the SSP/ISP force vector was fixed instead of the sole supraspinatus tendon as formerly described [3, 17]. This was performed due to the described interdigitation of infraspinatus (ISP) and supraspinatus (SSP) fibres [7] with overlapping of the fibres [21] at the humeral footprint. Furthermore an extend of the infraspinatus` footprint along most of the highest facet of the greater tuberosity [22] is reported [19].

In addition FibreTape instead of FibreWire was used for suture anchor material that could also be responsible for the different failure loads although Ishak et al. [11] reported on equivalent load capacity. Nevertheless single SR reconstruction is an important mode of fixation. However, it may result in over reduction of the bone fragment distally. The major advantage of the double row technique is that the bone fragment is fixed at the osteoarticular junction and avoids over reduction of the fragment.

In clinical practice significantly softer bone stock is sometimes seen medially to the fracture. If there are concerns regarding the stability of the medial row of the double row reconstruction, single row fixation represents a high quality alternative but with biomechanically reduced load to failure. One of the major advantages of this suture technique is that fixation depends on the strongest tissue in the area which is the rotator cuff tendon itself.

Besides, in case of comminuted fractures of the greater tuberosity or in osteopenic bone, this study could provide important information regarding suture anchor reconstruction that underlines the clinical relevance of our study.

### Limitations

There are several limitations to this study that need to be considered. First, using an in-vitro cadaveric model, the study can only assess direct postoperative (time-zero) stability, not accounting for any biological influences during the healing period.

In addition, the influence of abduction, elevation or rotation and its shear, compression, or torsion forces to the fixation construct were not assessed what may affect the clinical situation. However, used postero-lateral force of SSP and ISP is closer to clinically reality than previous setups.

Also, the artificially induced fractures of the greater tuberosity may not reflect completely the conditions in vivo as fractures of the greater tuberosity are often comminuted. However, comminuted fractures are difficult to induce and reproduce artificially.

In addition, the influence of poor bone quality on the fixation stability was not assessed. Furthermore, although sample size is comparable to existing studies [3, 17] focussing on this topic, the study may be underpowered.

## Conclusion

In this biomechanical study arthroscopic single- and double-row suture anchor repair of isolated greater tuberosity fractures seem to be viable options for treatment. Therefore, in the setting of reduced bone quality and weak anchorage of the medial row anchors, the authors perform a single row repair in daily clinical practice.

## Abbreviations

AF: Anchor fixation
DR: Double row
GT: Greater Tuberosity
H: Humerus
HH: Humeral Head
ISP: Infraspinatus
LA: Lateral anchors
MA: Medial anchors
PSRC: Postero-superior rotator cuff
SR: Single Row
SSP: Supraspinatus
